# Trastuzumab-TKI combination in HER2-Positive tumors: a FAERS-Based safety profile and multimodal analysis of Tanespimycin’s role in enhancing targeting of the HSP90AA1-PI3K-Akt-mTOR axis

**DOI:** 10.3389/fphar.2026.1844013

**Published:** 2026-08-26

**Authors:** Xiao Chen, Xiao Han, Yuanyuan Zhang, Junming Cao, Xin Wang

**Affiliations:** The First Department of Breast Cancer, Tianjin’s Clinical Research Center for Cancer, Key Laboratory of Breast Cancer Prevention and Therapy, Tianjin Medical University Cancer Institute & Hospital, National Clinical Research Center for Cancer, Key Laboratory of Breast Cancer Prevention and Therapy, Tianjin Medical University, Ministry of Education, Tianjin, China

**Keywords:** FAERS, Her2-positive tumors, HSP90AA1, lapatinib, PI3K/AKT/mTOR, tanespimycin, trastuzumab

## Abstract

**Background:**

This study addresses the safety profiles and molecular mechanisms of trastuzumab monotherapy and its combination with lapatinib, neratinib, and tucatinib for treating HER2-positive tumors.

**Methods:**

The research integrates a multi-platform approach, including data from the US Food and Drug Administration Adverse Event Reporting System (FAERS), network pharmacology, molecular docking, molecular dynamics simulations, and *in vitro* experiments. Specifically, 38,300 FAERS reports were screened, alongside a series of *in silico* modeling and laboratory assays to systematically evaluate drug safety and therapeutic mechanisms.

**Results:**

Pharmacovigilance analysis revealed that trastuzumab monotherapy was associated with an elevated incidence of cardiac and tumor progression-related adverse events (AEs). Combination therapies showed longer adverse event latency periods. Network pharmacology identified HSP90AA1 as a pivotal therapeutic target, while molecular docking and 100-ns molecular dynamics simulations confirmed the stable binding of each drug to HSP90AA1. *In vitro* assays demonstrated that HSP90AA1 is significantly overexpressed in breast cancer, driving cell proliferation and invasion, and correlates with a poor prognosis. Furthermore, the triple regimen of trastuzumab, lapatinib, and tanespimycin significantly inhibited the PI3K/Akt/mTOR signaling pathway.

**Conclusion:**

This study establishes a novel framework for drug safety evaluation and provides a theoretical rationale for optimizing therapeutic strategies in HER2-positive tumors. These findings highlight the potential advantages of combination therapies regarding AE latency and elucidate the critical role of HSP90AA1.

## 1 Introduction

Breast cancer remains the most prevalent malignancy in women globally, representing a substantial public health burden due to its high morbidity and mortality (Bray et al., 2024). Among the heterogeneous subtypes of breast cancer, human epidermal growth factor receptor 2 (HER2)-positive cases constitute approximately 15%–20% of the population, defined by HER2 protein overexpression or gene amplification (Guo et al., 2021). Historically, this subtype was correlated with an aggressive clinical phenotype and an unfavorable prognosis, presenting significant therapeutic hurdles (Yang et al., 2022). However, the advent of targeted anti-HER2 monoclonal antibodies, particularly trastuzumab, has revolutionized the therapeutic landscape, yielding marked improvements in survival outcomes for patients with HER2-positive disease (Cordero et al., 2022).

Despite the clinical success of trastuzumab, monotherapy is frequently hampered by primary or acquired resistance, as well as toxicity-induced treatment discontinuation, ultimately compromising disease control (Ande et al., 2018; Grela-Wojewoda et al., 2022). To circumvent these hurdles, novel HER2-targeted small-molecule tyrosine kinase inhibitors (TKIs), specifically lapatinib, neratinib, and tucatinib, have been introduced as combinatorial partners with trastuzumab. Mechanistically, these agents induce antitumor activity by abrogating downstream signaling cascades associated with HER2 and related family members, such as EGFR (Hino et al., 2022; Collins et al., 2021). While lapatinib and neratinib function as pan-HER inhibitors, tucatinib is distinguished by its high selectivity for HER2, demonstrating profound efficacy, particularly in the management of brain metastases (Yamamoto et al., 2022). Clinical evidence has substantiated the superior therapeutic index of these combinatorial regimens over monotherapy, establishing them as the standard of care for advanced HER2-positive disease.

However, the widespread clinical adoption of combination therapies has introduced new complexities in managing associated adverse events (AEs). The co-administration of multiple agents often results in distinct toxicity profiles and risk stratifications that may elude detection within the controlled, finite confines of pre-marketing clinical trials. Notably, pan-HER inhibitors are frequently implicated in severe dermatologic and gastrointestinal toxicities (Liu et al., 2019). Consequently, a systematic investigation into the real-world safety profiles of these regimens, including the spectrum, frequency, and onset kinetics of AEs, is imperative for optimizing clinical decision-making and safeguarding patient welfare. While randomized controlled trials (RCTs) provide standardized safety data, their stringent inclusion criteria often restrict the generalizability of findings to the diverse patient populations encountered in routine clinical practice (Abuhelwa et al., 2022). In this context, the FDA Adverse Event Reporting System (FAERS), one of the world’s largest post-marketing pharmacovigilance repositories, serves as a critical resource for evaluating drug-related AEs in a real-world setting. Leveraging data mining and signal detection algorithms allows for the identification of potential safety signals, facilitating the detection of rare or delayed AEs often previously unobserved in pre-marketing studies (Hu et al., 2022).

Complementing the clinical safety evaluation, elucidating the shared molecular mechanisms governing these combinatorial regimens is of critical importance. While these agents possess distinct targets, they collectively mediate therapeutic efficacy by inhibiting HER2 signaling to suppress tumor growth (Song et al., 2021). However, tumor cells frequently acquire resistance via the activation of downstream compensatory signaling, notably the PI3K/Akt/mTOR and MAPK cascades (Zhao M. et al., 2021). Consequently, determining whether these regimens mediate synergy through shared molecular targets or pathways is a prerequisite for optimizing therapeutic strategies. Network pharmacology, as a systems biology approach, enables comprehensive dissection of complex drug mechanisms through the construction of drug-target-pathway networks, thereby facilitating the identification of potential core therapeutic targets (Xue et al., 2022). Furthermore, computational approaches including molecular docking and molecular dynamics simulations validate the binding affinity and stability between pharmaceutical agents and target proteins at the atomic level, providing a robust structural basis for mechanistic investigations (Gom et al., 2022).

Crucially, there is a fundamental need to seamlessly bridge the clinical toxicity profiles with the underlying molecular pharmacology. While clinical use of TKIs effectively halts tumor progression, it concurrently induces severe dose-dependent and off-target adverse events, such as the high incidence of diarrhea observed in pharmacovigilance data. Therefore, identifying a distinct, unifying molecular hub (such as a shared molecular chaperone) that is essential for tumor survival could provide a vital pharmacological workaround. We hypothesize that by concurrently targeting such a shared node, we can achieve profound synergistic efficacy. This synergy could theoretically enable therapeutic efficacy at reduced TKI doses (a dose-sparing effect), thereby directly mitigating the specific clinical toxicities identified in our FAERS analysis without sacrificing anti-tumor potency.

This study integrates the aforementioned methodologies to conduct a comprehensive pharmacological and safety assessment of trastuzumab combined with lapatinib, neratinib, and tucatinib. Our specific objectives are (Bray et al., 2024): to conduct a systematic comparative analysis of adverse event profiles, time-to-onset, and risk stratification for distinct combinatorial regimens using the FAERS database (Guo et al., 2021); to utilize network pharmacology to elucidate shared pivotal targets implicated in tumor progression across these pharmaceutical agents at a systems level (Yang et al., 2022); to characterize the binding interactions between key protein targets and ligands via molecular docking and molecular dynamics simulations; and (Cordero et al., 2022) to validate the clinical significance of identified targets and their modulation of critical signaling pathways through integrated bioinformatics analysis and *in vitro* validation. We hypothesize that while exhibiting distinct safety profiles, these combinatorial regimens achieve synergistic antitumor efficacy by converging upon critical molecular chaperones, notably HSP90AA1, thereby curtailing tumor proliferation and abrogating resistance pathways. By employing this multidimensional framework, we aim to establish a mechanistic and pharmacological proof-of-concept, providing new insights into optimizing treatment strategies for HER2-positive breast cancer and laying a theoretical foundation for overcoming drug resistance. During the preparation of this work, the authors did not use any generative AI technologies (Agha et al., 2025).

## 2 Materials and methods

### 2.1 FAERS

#### 2.1.1 Study design and data sources

This retrospective, observational pharmacovigilance study utilized data derived from the US Food and Drug Administration (FDA) Adverse Event Reporting System (FAERS). Maintained by the FDA, this repository serves as a primary instrument for post-marketing surveillance to monitor the safety profiles of pharmaceutical agents and therapeutic biologics. By aggregating global reports regarding adverse events (AEs) and medication errors, the system facilitates the identification of potential safety signals.

#### 2.1.2 Procedures

The FAERS database was queried to retrieve adverse event (AE) reports associated with four therapeutic agents: trastuzumab, lapatinib, neratinib, and tucatinib. Comprehensive demographic and clinical data were extracted, encompassing sex, body weight, age, clinical outcomes, reporter profession, and reporting country. All adverse events were systematically coded using Preferred Terms (PTs) from the Medical Dictionary for Regulatory Activities (MedDRA). Duplicate entries were identified and eliminated to ensure data integrity. Subsequently, cases were stratified into four cohorts based on the medication regimen: trastuzumab monotherapy, and trastuzumab combined with lapatinib, neratinib, or tucatinib.

#### 2.1.3 Statistical analysis

Disproportionality analysis constitutes a pivotal data mining technique employed to detect safety signals concerning adverse drug reactions (ADRs). This method quantifies potential associations between pharmaceutical agents and adverse events (AEs) by mining large scale pharmacovigilance repositories. Standard algorithms utilized in this domain encompass the Information Component (IC), Reporting Odds Ratio (ROR), Proportional Reporting Ratio (PRR), Multi-item Gamma Poisson Shrinker (MGPS), and Bayesian Confidence Propagation Neural Network (BCPNN). Statistical significance was defined when the lower bound of the 95% confidence interval for the IC (IC025) exceeded zero. These algorithms (particularly IC and ROR) were specifically selected because they inherently normalize for sample size discrepancies by comparing observed-to-expected ratios rather than raw frequencies. This ensures statistical robustness even when comparing treatment groups with vastly different total report counts (e.g., monotherapy vs. combination).

In the FDA Adverse Event Reporting System (FAERS), Time to Onset (TTO) and Weibull Shape Parameter (WSP) are two critical metrics for evaluating the temporal characteristics and risk patterns of adverse events (AEs). TTO quantifies the latency period of an adverse event, revealing the temporal relationship between drug exposure and AE occurrence. WSP, derived from the Weibull distribution model, is a statistical parameter used to analyze the temporal distribution of AEs. The Weibull distribution is defined by: λ (scale parameter): Reflects the average time to AE occurrence. β (shape parameter): Determines the temporal pattern of AE risk: β < 1 and the 95% CI < 1, the AE follows an early failure pattern (risk decreases over time). β ≈ 1 and the 95% CI includes 1, the AE exhibits a random failure pattern (constant risk over time). β > 1 and the 95% CI > 1, the AE follows a wear-out failure pattern (risk increases over time). All statistical analyses were performed using R statistical software (version 4.5.0).

### 2.2 Network pharmacology, molecular docking, molecular dynamics simulation

#### 2.2.1 Data collection and target identification

Disease targets associated with tumor progression were collected from open-source databases, including GeneCards (https://www.genecards.org) and OMIM (https://omim.org). The potential targets of four therapeutic agents (trastuzumab, lapatinib, neratinib, and tucatinib) were predicted using GeneCards (https://www.genecards.org), SwissTargetPrediction (http://www.swisstargetprediction.ch/index.php), and DrugCentral 2023 (https://drugcentral.org/). Subsequently, a Venn diagram was constructed using Venny 2.1.0 (https://bioinfogp.cnb.csic.es/tools/venny/) to visualize overlapping targets among these drugs.

#### 2.2.2 Protein-protein interaction (PPI) network construction

The PPI network was constructed using the STRING online tool (version 12.0; https://cn.string-db.org/). Key parameters for PPI network construction were set as follows: organism was limited to *Homo sapiens*, and the minimum required interaction score was set to medium confidence (0.400). The resulting network was imported into Cytoscape (version 3.10.3; https://www.cytoscape.org/) for visualization and systematic topological analysis. Core targets were identified through cluster analysis using the CytoNCA plugin in Cytoscape. The protein-protein interaction (PPI) network was filtered using the Molecular Complex Detection (MCODE) plugin in Cytoscape, with the following parameters: degree cutoff = 2, k-core = 2, node score cutoff = 0.2, and max depth = 100. Additionally, cross-validation was performed using the cytoHubba plugin with multiple algorithms, with particular emphasis on the Maximal Clique Centrality (MCC) method due to its superior capability in identifying biologically relevant hub genes.

#### 2.2.3 Drug‐Target‐Pathway network

The overlapping targets and bioactive compounds were integrated into Cytoscape (version 3.10.3) to establish a comprehensive Drug-Target-Pathway (DTP) network. Topological analysis was subsequently performed using the built-in ‘Network Analyzer’ tool to evaluate key network parameters.

#### 2.2.4 Molecular docking

The protein structure was pre-processed using PyMOL (version 2.6) to remove water molecules and native ligands from the crystal structure in preparation for molecular docking analysis. The processed protein was then imported into AutoDockTools (version 1.5.7) for hydrogen addition, charge calculation, charge assignment, and atom type designation before final structure optimization. The five candidate drugs underwent identical preparation steps in AutoDockTools to ensure structural compatibility. Molecular docking simulations were performed using AutoDock Vina to predict binding modes and affinity between the ligands and HSP90AA1. Post-docking analysis was conducted using PyMOL and Open Babel GUI (version 3.1.1) for detailed visualization and interaction pattern assessment.

#### 2.2.5 Molecular dynamic simulation

In a synthetic setting, molecular dynamics (MD) simulation aids in identifying and verifying the entropic effects and structural flexibility of protein–ligand complexes. We selected Trastuzumab-HSP90AA1 complex, Lapatinib-HSP90AA1 complex, Neratinib-HSP90AA1 complex, Tucatinib-HSP90AA1 complex and Tanespimycin-HSP90AA1 complex for MD simulations. The investigations were conducted utilizing the GROMACS 2023.2 software package (Abraham et al., 2015). The simulation system was set up in a dodecahedron box, utilizing the Amber99s-protein force field. All components were solvated in the TIP3P water model, and Na+ and Cl− ions were added to neutralize the system charge (Zheng et al., 2025). The energy minimization (EM) phase used the steepest descent integrator, terminating when the maximum force dropped below 10.0 kJ/mol. Following energy minimization, the setup underwent thermal adjustment for 100 ps at 300 K within an NVT ensemble utilizing periodic boundary parameters. The simulation employed a 2 fs timestep, with hydrogen-containing covalent bonds constrained through LINCS protocol. A V-rescale thermostat maintained the 300 K temperature, while PME methodology handled electrostatic forces with a 1.0 nm cutoff. The process included a brief 100 ps equilibration phase, succeeded by main simulations utilizing 2 fs steps in an NPT ensemble under identical protocols. The Parrinello-Rahman algorithm regulated pressure at 1.0 atm. The final simulation run was conducted for 100 ns at 300 K and 1 atm for all the simulation systems. The resulting MD simulation trajectories were analyzed to observe key metrics such as the Root Mean Square Deviation (RMSD), Root Mean Square Fluctuation (RMSF), radius of gyration (Rg), and solvent accessible surface areathe (SASA), and hydrogen bonding using GROMACS functions (gmx_rmsd, gmx_rms, gmx_gyrate, gmx_sasa, and gmx_hbound). All xvg files resulting from these procedures were visualized using the qtGrace software (https://sourceforge.net/projects/qtgrace/).

### 2.3 GO and KEGG enrichment analyses

The selected genes were subjected to Gene Ontology (GO) and Kyoto Encyclopedia of Genes and Genomes (KEGG) pathway analyses using the DAVID database (version 6.8; https://david.ncifcrf.gov/). The GO analysis encompassed three categories: cellular component (CC), molecular function (MF), and biological process (BP). Statistical significance thresholds were set at false discovery rate (FDR) < 0.05 and P-value < 0.05. Visualization of the results was performed using R statistical software (version 4.5.0).

### 2.4 Survival and expression analysis of HSP90AA1 in breast cancer

The prognostic significance of HSP90AA1 in breast cancer was evaluated using the KM Plotter database (https://kmplot.com/analysis/) to obtain survival data. Clinical stage-associated expression patterns of HSP90AA1 were analyzed via the GEPIA database (http://gepia.cancer-pku.cn/), which also provided differential expression profiles between normal breast tissue and breast tumor samples. Protein-level validation was performed using immunohistochemical (IHC) images from the Human Protein Atlas (HPA, https://www.proteinatlas.org/), comparing HSP90AA1 expression in normal versus malignant tissues.

### 2.5 Cell culture, plasmids, and transfection

The HCC-1954 cell line was obtained from Pricella (China), which provided certification of authentication via Short Tandem Repeat (STR) profiling. HCC-1954 were cultured in 1,640 (Gibco, Grand Island, USA). All medium included 10% fetal bovine serum (FBS, NEWZERUM, Australia) and 1% penicillin/streptomycin (Gibco, Grand Island, USA). The BT474 cell line was obtained from Shanghai Puhongnuo Biotechnology Co., Ltd., which provided certification of authentication via Short Tandem Repeat (STR) profiling. BT474 were cultured in 1,640 (Gibco, Grand Island, USA). All medium included 10% fetal bovine serum (FBS, NEWZERUM, Australia), 1% penicillin/streptomycin (Gibco, Grand Island, USA) and insulin (10ug/mL, Pricella, China). Cells were cultured at 37 °C in a humidified cell incubator with a 5% CO2 atmosphere. The HSP90AA1 plasmids and corresponding vectors were purchased from GeneCopoeia (Guangzhou, China). Transfection was performed using Lipofectamine 3,000 (Invitrogen, California, USA).

### 2.6 Proliferation assay

For colony formation analysis, 700 cells were seeded per well in six-well plates and cultured until colonies had developed to an appropriate size. Subsequently, the colonies were fixed with 4% paraformaldehyde and stained with 1% crystal violet. The stained colonies were imaged and quantified manually. Cell proliferation was assessed using the 5-ethynyl-2′-deoxyuridine (EdU) assay kit (RiboBio, China) in accordance with the manufacturer’s protocol. The percentage of EdU-positive cells was determined based on fluorescence microscopy (Leica, Germany) and analyzed using appropriate image processing software.

### 2.7 Cell migration and invasion assays

Cell invasion was assessed using Matrigel-coated Transwell inserts (BD Biosciences, New Jersey, USA). Briefly, 60,000 cells suspended in serum-free medium were plated in the upper chamber, while the lower chamber was filled with medium supplemented with 20% FBS as a chemoattractant. After incubation for 16–24 h, cells that had invaded through the Matrigel were fixed and stained using a three-step staining kit (Thermo Scientific, Massachusetts, USA). The number of invaded cells was quantified by counting under a light microscope (Olympus, Japan) at ×100 magnification.

For the wound healing assay, cells were cultured in 6-well plates until they reached full confluence. A uniform wound was created in each well using a sterile 10 µL pipette tip. The cells were then washed and maintained in serum-free medium to eliminate the potential influence of serum on cell migration. Wound images were captured at 0 and 48 h using a light microscope (Olympus, Japan). The extent of migration was evaluated by measuring the change in wound area relative to the initial time point (0 h) with image analysis software.

### 2.8 Western blot analysis

Proteins extracted from cell lysates were separated by SDS-PAGE and subsequently transferred onto polyvinylidene fluoride (PVDF) membranes. The membranes were blocked and then incubated with specific primary antibodies against the target proteins, followed by incubation with appropriate horseradish peroxidase (HRP)-conjugated secondary antibodies. Protein bands were visualized using an enhanced chemiluminescence (ECL) detection reagent (Millipore, Bedford, MA, USA). Signal intensities were quantified using ImageJ software (National Institutes of Health, USA), and the values were normalized to the corresponding loading controls.

### 2.9 TUNEL staining

The level of cell apoptosis was evaluated using the One-step TUNEL *In Situ* Apoptosis Kit (Elabscience). After treatment with specific drugs, cells were washed with PBS and fixed with 4% paraformaldehyde for 15 min at room temperature. Following PBS washing, cells were permeabilized with 0.2% Triton X-100 for 10 min at room temperature. Subsequently, TdT Equilibration Buffer was added to each well and incubated at 37 °C for 10–30 min. After removing the equilibration buffer, freshly prepared TUNEL labeling working solution (containing TdT Enzyme and Labeling Solution) was added, and the cells were incubated for 60 min at 37 °C in the dark. Upon completion of the reaction, cells were thoroughly washed with PBS to remove unbound labels, and then counterstained with DAPI working solution for 5 min at room temperature in the dark. Finally, cells were observed and images were captured using a fluorescence microscope.

### 2.10 Antibodies and drugs

Antibodies against Akt (cat. No. 10176-2-AP) and GAPDH (cat. No. 60004-1-Ig) were obtained from Proteintech. The β-actin antibody (cat. No. YM8343) was acquired from Immunoway. PI3K (cat. No. 4249), Phospho-PI3K p85 (Tyr458; cat. No. 4228), Phospho-Akt (Ser473; cat. No. 13038S), phospho-mTOR (Ser2448; cat. No. 5536S), mTOR (cat. No. 2983S), Bax (cat. No. 2772), Bcl-XL (cat. No. 2764), PARP (cat. No. 9532), Cleaved PARP (cat. No. 5625) and α-tubulin (cat. No. 3873) antibodies were purchased from Cell Signaling Technology. Bcl-2 (Catalog# ET1702-53) and Cleaved + pro Caspase-3 (Catalog# ET1608-64) were purchased from HUABIO. The antibody targeting HSP90AA1 (cat. No. HY-P83995) was sourced from MedChemExpress. The inhibitors trastuzumab (cat. No. HY-P9907), lapatinib (cat. No. HY-50898A), paclitaxel (cat. No. HY-B0015), pertuzumab (cat. No. HY-P9912) and tanespimycin (cat. No. HY-10211) were also procured from MedChemExpress.

For the combination assays, HCC-1954 cells were seeded and allowed to attach overnight. The cells were then treated with Trastuzumab (5 μg/mL) and Tanespimycin (1 µM) for a total duration of 24 h. Lapatinib (5 µM) was added to the culture medium for the final 6 h of the treatment period. The concentrations of Paclitaxel and Pertuzumab were 10 nM and 5 μg/mL, respectively, for a 24-h incubation period.

### 2.11 Statistical analysis

All experiments were performed in triplicate and independently repeated at least three times. Data are presented as mean ± standard deviation (SD). Statistical analyses were carried out using GraphPad Prism software (version 5.0). Differences among multiple groups were assessed by one-way analysis of variance (ANOVA) with appropriate *post hoc* tests for multiple comparisons. Comparisons between two independent groups were conducted using Student’s t-test. A P-value of less than 0.05 was considered statistically significant.

## 3 Results

### 3.1 FAERS

#### 3.1.1 Baseline characteristics of patients

Following the exclusion of duplicate and invalid entries, detailed patient characteristics are summarized (Table 1). The final cohort comprised 35,327 patients receiving trastuzumab monotherapy, while those treated with trastuzumab combined with lapatinib, neratinib, or tucatinib numbered 1,053, 154, and 1,766, respectively. Females constituted the predominant population across all cohorts. Regarding age distribution, the subgroup aged 45–64 years represented the largest proportion. Physicians accounted for the majority of reporters. Notably, the highest mortality rate (28.57%) was recorded in the cohort treated with trastuzumab and neratinib, whereas the highest hospitalization rate (24.92%) was observed in the group receiving trastuzumab combined with tucatinib. The United States contributed the most reports for all groups. Breast cancer remained the most frequently reported indication across all regimens, with metastatic disease frequently specified in the neratinib combination cohort.

**TABLE 1:** Clinical characteristics in different groups.

Characteristics	Trastuzumab	Trastuzumab+ Lapatinib	Trastuzumab+ Neratinib	Trastuzumab+ Tucatinib
Sex				
Female	26,241 (74.28%)	961 (91.26%)	78 (50.65%)	1,487 (84.20%)
Male	2,196 (6.22%)	15 (1.42%)	2 (1.30%)	87 (4.93%)
Unknown	6,890 (19.50%)	77 (7.31%)	74 (48.05%)	192 (10.87%)
Weight(kg)				
<50	992 (2.81%)	53 (5.03%)	—	42 (2.38%)
50–100	8,047 (22.78%)	309 (29.34%)	18 (11.69%)	318 (18.01%)
>100	420 (1.19%)	14 (1.33%)	1 (0.65%)	27 (1.53%)
Missing	25,868 (73.22%)	677 (64.29%)	135 (87.66%)	1,379 (78.09%)
Age				
≤18	34 (0.10%)	1 (0.10%)	—	5 (0.28%)
19–44	3,466 (9.81%)	122 (11.59%)	13 (8.44%)	172 (9.74%)
45–64	10,236 (28.98%)	450 (42.74%)	40 (25.97%)	452 (25.59%)
65–74	3,716 (10.52%)	133 (12.63%)	11 (7.14%)	189 (10.70%)
≥75	1,643 (4.65%)	49 (4.65%)	5 (3.25%)	59 (3.34%)
Missing	16,232 (45.95%)	298 (28.30%)	85 (55.19%)	889 (50.34%)
Reporter type				
Consumer	6,363 (18.01%)	501 (47.58%)	15 (9.74%)	357 (20.22%)
Pharmacist	2,994 (8.48%)	33 (3.13%)	11 (7.14%)	74 (4.19%)
Physician	16,193 (45.84%)	388 (36.85%)	80 (51.95%)	1,008 (57.08%)
Lawyer	20 (0.06%)	—	—	—
Health-professional	9,388 (26.57%)	123 (11.68%)	47 (30.52%)	327 (18.52%)
Unknown	369 (1.04%)	8 (0.76%)	1 (0.65%)	—
Outcome				
Death	5,138 (14.54%)	122 (11.59%)	44 (28.57%)	148 (8.38%)
Life-Threatening	1,188 (3.36%)	29 (2.75%)	5 (3.25%)	18 (1.02%)
Hospitalization	7,386 (20.91%)	149 (14.15%)	30 (19.48%)	440 (24.92%)
Disability	518 (1.47%)	5 (0.47%)	—	14 (0.79%)
Congenital anomaly	49 (0.14%)	1 (0.09%)	—	—
Required intervention	83 (0.23%)	1 (0.09%)	—	—
Others	14,767 (41.80%)	341 (32.38%)	51 (33.12%)	668 (37.83%)
Missing	6,198 (17.54%)	405 (38.46%)	24 (15.58%)	478 (27.07%)
Top five countries reported				
​	United States	United States	United States	United States
​	China	Canada	United Kingdom	France
​	Canada	Japan	Canada	Greece
​	United Kingdom	Australia	Austria	Germany
​	Japan	Austria	China	Austria
Top-ranked indication				
​	Breast cancer	Breast cancer	Breast cancer metastatic	Breast cancer

#### 3.1.2 Statistical analysis of adverse events across treatment groups

Adverse events (AEs) were compared among treatment groups using System Organ Class (SOC) categorization. In the trastuzumab monotherapy cohort, “Neoplasms benign, malignant and unspecified (incl cysts and polyps)” constituted the predominant SOC (n = 89; Table 2), followed by “Investigations” (n = 63), “Cardiac disorders” (n = 53), and “Infections and infestations” (n = 30). Interestingly, the monotherapy-dominant AE (neoplasms) exhibited lower frequencies in combination groups. Conversely, the primary toxicities observed in regimens combining trastuzumab with lapatinib shifted toward “Skin and subcutaneous tissue disorders” (n = 12), “Gastrointestinal disorders” (n = 7), and “Nervous system disorders” (n = 3), respectively. Overall, the aggregate incidence of reported AEs in combination therapies was substantially lower compared to monotherapy.

**TABLE 2:** System organ classes (SOCs) for adverse events of different groups.

SOC	Trastuzumab	Trastuzumab + Lapatinib	Trastuzumab + Neratinib	Trastuzumab + Tucatinib
Neoplasms benign, malignant and unspecified (incl cysts and polyps)	89	2	2	2
Investigations	63	0	2	6
Cardiac disorders	53	0	0	0
Infections and infestations	30	2	1	6
General disorders and administration site conditions	27	2	4	3
Respiratory, thoracic and mediastinal disorders	27	0	2	2
Skin and subcutaneous tissue disorders	25	12	3	7
Nervous system disorders	23	3	4	9
Injury, poisoning and procedural complications	22	1	5	2
Blood and lymphatic system disorders	19	0	2	2
Hepatobiliary disorders	17	0	1	5
Gastrointestinal disorders	16	7	6	7
Reproductive system and breast disorders	14	0	0	0
Surgical and medical procedures	14	1	0	1
Vascular disorders	14	0	0	0
Musculoskeletal and connective tissue disorders	11	0	0	0
Eye disorders	9	0	1	1
Congenital, familial and genetic disorders	6	0	0	1
Metabolism and nutrition disorders	5	1	3	0
Immune system disorders	2	0	0	0
Renal and urinary disorders	2	0	1	0
Endocrine disorders	1	0	0	2
Pregnancy, puerperium and perinatal conditions	1	0	0	0
Psychiatric disorders	1	0	1	2
Product issues	0	1	0	0
Social circumstances	0	0	0	1

#### 3.1.3 Time-to-onset (TTO) and Weibull Shape Parameter (WSP) analysis

The median TTO for trastuzumab monotherapy was 26 days (IQR: 4–147; Table 3). All combinatorial regimens exhibited prolonged median TTOs compared to monotherapy, with the combination of trastuzumab and neratinib demonstrating the most extended latency (92 days; IQR: 27–721). The remaining two combination regimens displayed comparable TTO profiles. WSP analysis revealed shape parameters (β) <1 with 95% CIs excluding 1 for all groups, indicating early failure-type patterns (i.e., risk concentration in initial treatment phases). Compared to monotherapy (β = 0.579), trastuzumab-lapatinib showed a lower β (0.488), suggesting enhanced early risk, whereas neratinib (β = 0.634) and tucatinib (β = 0.614) combinations exhibited relatively attenuated early risks.

**TABLE 3:** TTO and WSP analysis of different groups.

Drug	TTO (days)		Weibull distribution		​
	Case reports	Median(d) (IQR)	Scale parameter α (95%CI)	Shape parameter Β (95%CI)	Type
Trastuzumab +Lapatinib	386	41.5 (3–352.5)	203.18 (156.28–250.08)	0.488 (0.447–0.528)	Early failure
Trastuzumab +Neratinib	59	92 (27–721)	304.67 (171.96–437.39)	0.634 (0.500–0.768)	Early failure
Trastuzumab +Tucatinib	503	42 (14–123)	114.26 (96.37–132.16)	0.614 (0.574–0.655)	Early failure
Trastuzumab	11,499	26 (4–147)	122.51 (117.98–127.04)	0.579 (0.570–0.588)	Early failure

#### 3.1.4 AE spectrum disparities among treatment groups

**FIGURE 1:**
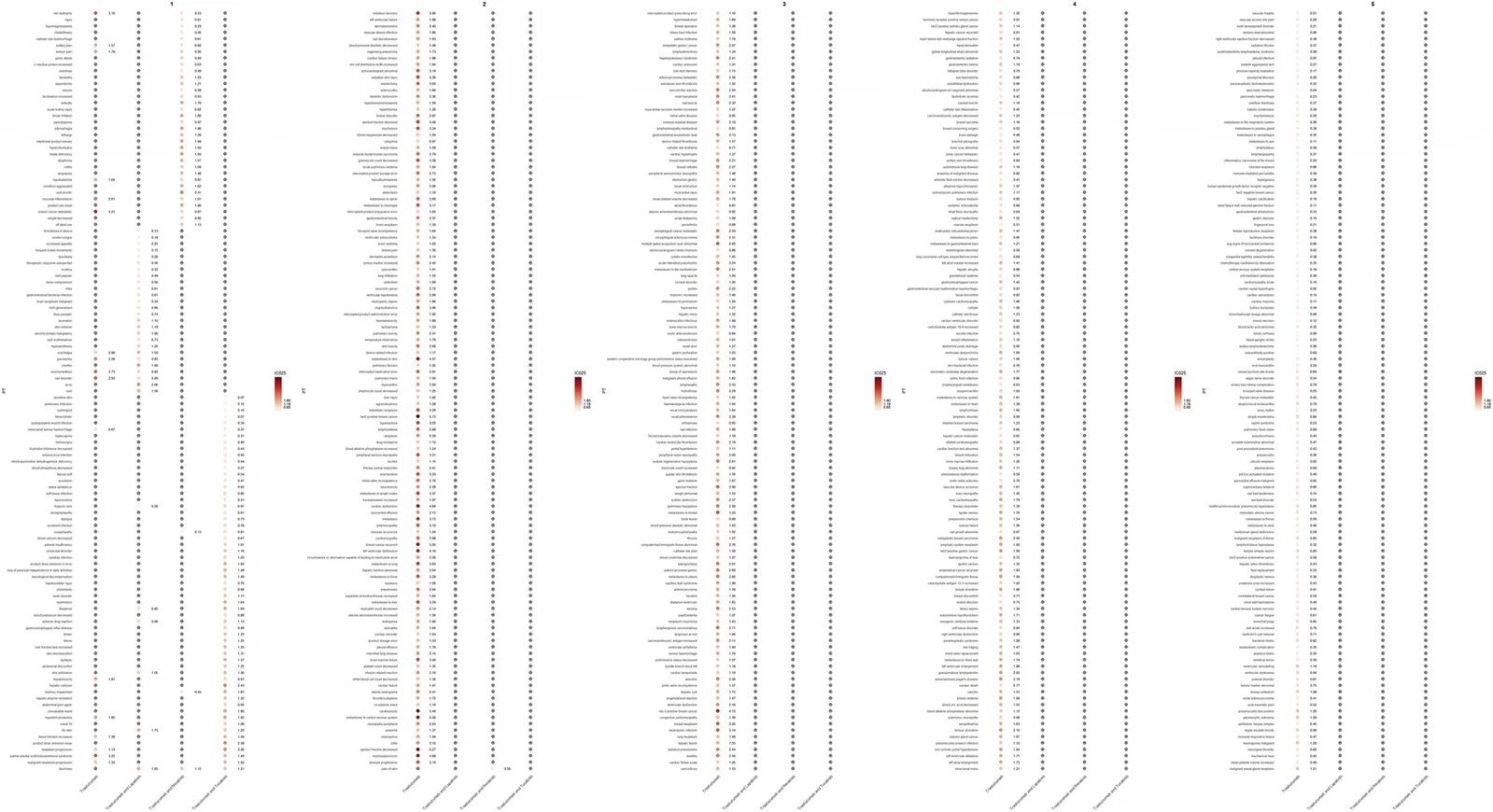
PT for four different therapy strategies. In Figure 1, PT: preferred term; IC: information component; IC025: the lower end of the 95% confidence interval of IC. IC025 greater than 0 was deemed a signal.

Figure 1 illustrates distinct AE profiles across groups, with signals defined as IC025 (lower bound of IC’s 95% CI) > 0. Minimal overlap was observed among groups. Trastuzumab monotherapy exhibited the most extensive AE spectrum, ranging from “Cardiac septal hypertrophy” (IC025 = 0.02) to “HER2 positive breast cancer” (IC025 = 5.73). Other significant Preferred Terms (PTs) associated with monotherapy (IC025 ≥ 5) included “Breast cancer recurrence”, “Cardiotoxicity”, “CNS metastases”, and “Decreased ejection fraction”, which primarily reflect oncologic progression and cardiac toxicity. The regimen combining trastuzumab and lapatinib presented 32 significant PTs (ranging from “Thrombosis in device” [IC025 = 0.13] to “Acne” [IC025 = 2.06]), whereas the combination of trastuzumab and neratinib was associated with 38 PTs, with “Pruritic rash” (IC025 = 2.41) representing the strongest signal. The cohort receiving trastuzumab and tucatinib yielded the greatest number of significant PTs among the combinatorial regimens, identifying “Hepatic cytolysis” (IC025 = 2.43) as the most prominent signal. Notably, “Diarrhea” was a signal exclusive to combination therapies, exhibiting the highest IC025 value in the lapatinib containing regimen (1.65).

### 3.2 Network pharmacology, molecular docking, molecular dynamics simulation

#### 3.2.1 Identification and analysis of therapeutic targets for tumor progression

**FIGURE 2:**
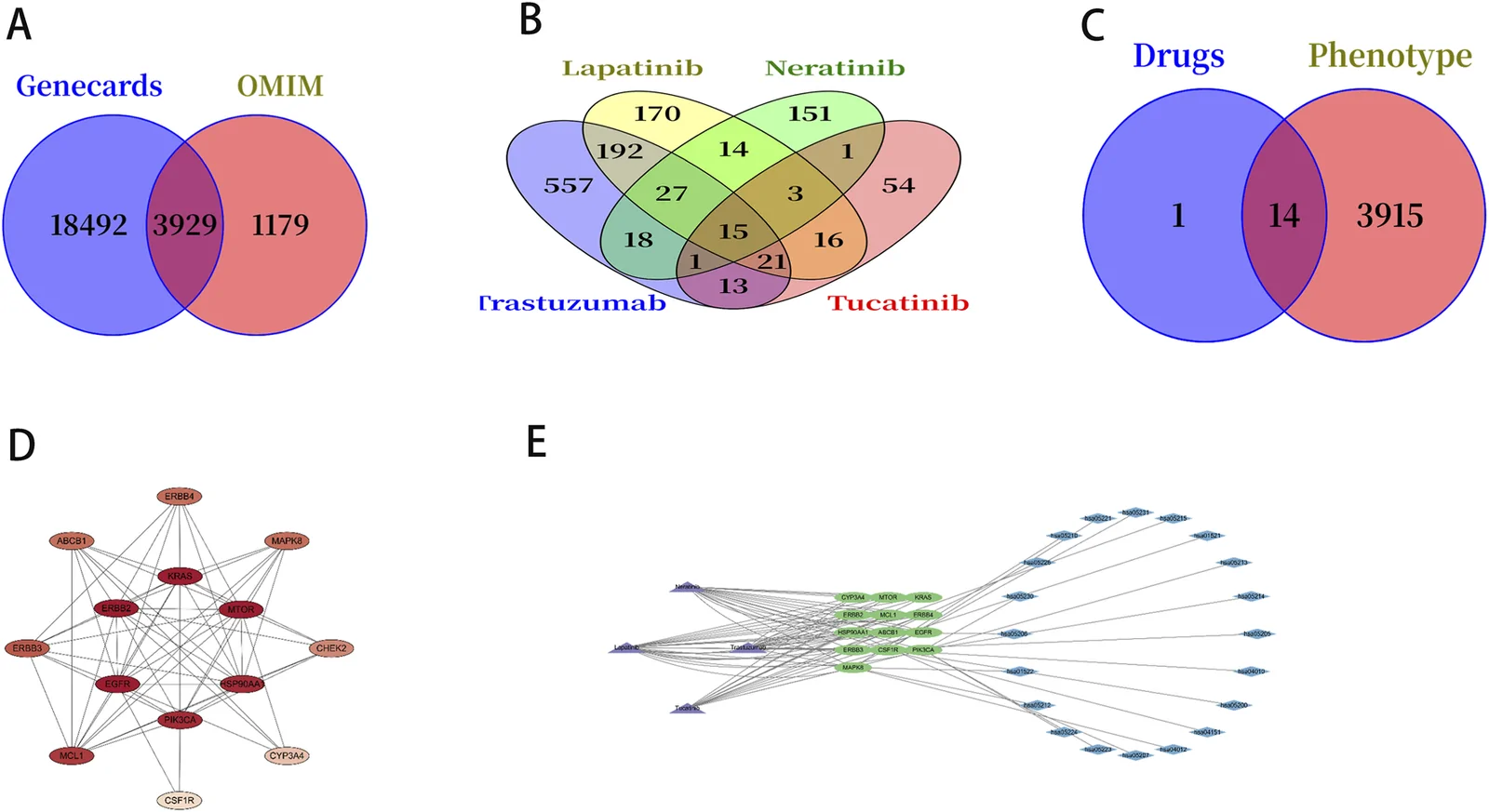
**(A)** Venn diagram of GeneCards and OMIM databases in tumor progression. **(B)** Venn diagram of four drugs. **(C)** Venn diagram of drugs and targets of tumor progression. **(D)** Protein-protein interaction network of common targets. **(E)** The network of Drug-Target-Pathway network.

Given that tumor progression constitutes the primary indication for all four therapeutic agents, potential targets were systematically identified via the GeneCards and OMIM databases (Figure 2A). Utilization of the Venny platform revealed 3,929 targets associated with tumor progression. Concurrently, 15 targets were found to be shared across all four pharmaceutical agents (Figure 2B). A subsequent intersection analysis between the 3,929 targets linked to tumor progression and the 15 targets specific to the drugs yielded 14 common candidates (Figure 2C).

These 14 shared targets were mapped onto the STRING database to construct a protein interaction network (PPI), which was subsequently visualized via Cytoscape. As shown in Figure 2D, the resulting PPI network comprised 14 nodes and 61 edges. Subsequent analysis using the MCODE algorithm identified 12 core genes, ranked in descending order of degree centrality: KRAS, ERBB2, EGFR, MTOR, HSP90AA1, PIK3CA, MCL1, ERBB3, ABCB1, MAPK8, ERBB4, and CHEK2 (Table 4). Notably, the top 12 candidate targets prioritized via Maximal Clique Centrality (MCC) scores demonstrated substantial concordance with the cluster identified by MCODE.

**TABLE 4:** Top 12 genes ranked by MCC method.

Rank	Name	Score
1	ERBB2	16,584
2	MTOR	16,584
3	KRAS	16,566
4	HSP90AA1	16,560
5	EGFR	15,870
6	PIK3CA	15,846
7	MCL1	11,520
8	ERBB3	10,080
9	MAPK8	5,040
10	ERBB4	5,040
11	ABCB1	744
12	CHEK2	720

#### 3.2.2 GO and KEGG analysis

**FIGURE 3:**
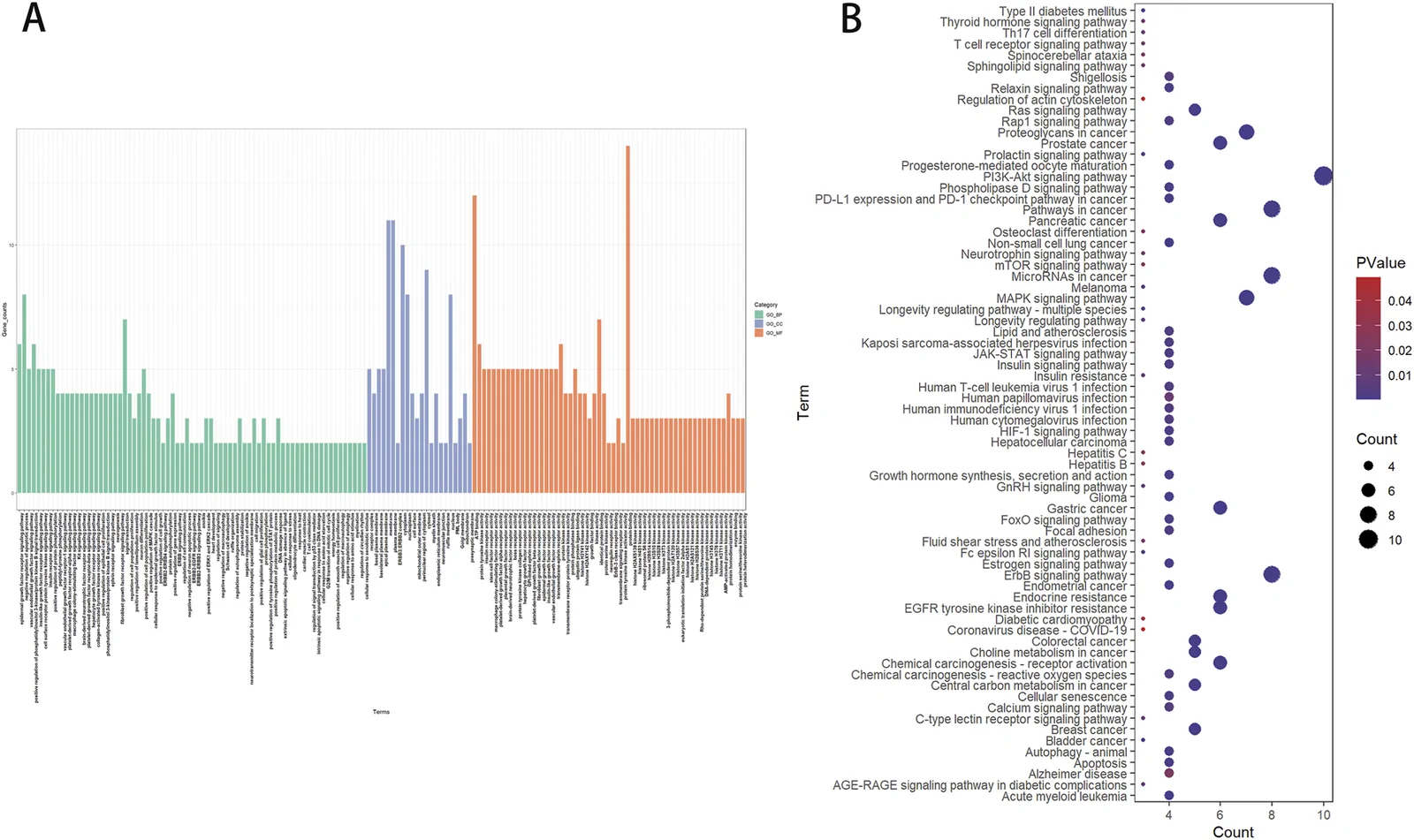
Analysis of GO functional enrichment **(A)** and KEGG pathway enrichment **(B)**.

Functional enrichment analysis was conducted to systematically characterize the biological significance of the potential therapeutic targets. Gene Ontology (GO) analysis identified 14 significantly enriched terms each for Biological Processes (BP), Cellular Components (CC), and Molecular Functions (MF) (Figure 3A). The BP analysis revealed predominant involvement in negative regulation of apoptotic processes, signal transduction, and epidermal growth factor receptor signaling pathways. CC analysis demonstrated primary localization to membrane structures (including plasma membrane) and cytoplasm, while MF analysis indicated crucial roles in protein binding, ATP binding, and identical protein binding. Collectively, these findings suggest that the identified targets modulate biological processes critical to tumor progression.

To further elucidate the signaling networks involved, KEGG pathway enrichment analysis was performed (Figure 3B). The results demonstrated significant enrichment in multiple oncogenic pathways, most notably the PI3K/Akt signaling pathway, ErbB signaling pathway, and MAPK signaling pathway. Crucially, analysis of pathways specific to distinct cancer types revealed involvement in breast cancer, gastric cancer, colorectal cancer, non-small cell lung cancer, pancreatic cancer, prostate cancer, endometrial cancer, acute myeloid leukemia, and glioma pathogenesis. These results strongly suggest that the screened target genes may contribute to tumor progression through modulation of these critical oncogenic signaling cascades and tumor-specific pathological mechanisms.

#### 3.2.3 Construction and analysis of the drug-target-pathway network

As illustrated in Figure 2E, we constructed a Drug-Target-Pathway network using Cytoscape by selecting the top 20 KEGG pathways (Table 5). The resulting network comprised 37 nodes and 87 edges, with each edge representing either drug-target or target-pathway interactions. In this network architecture, the degree value quantifies the number of edges connected to each node.

**TABLE 5:** Top 20 highly enriched pathways.

ID	Pathways	Count	PValue	Genes
hsa04151	PI3K-Akt signaling pathway	10	2.48E-10	CSF1R, HSP90AA1, ERBB3, PIK3CA, ERBB4, ERBB2, KRAS, EGFR, MTOR, MCL1
hsa04012	ErbB signaling pathway	8	1.35E-11	MAPK8, ERBB3, PIK3CA, ERBB4, ERBB2, KRAS, EGFR, MTOR
hsa05206	MicroRNAs in cancer	8	1.16E-07	ABCB1, ERBB3, PIK3CA, ERBB2, KRAS, EGFR, MTOR, MCL1
hsa05200	Pathways in cancer	8	4.40E-06	CSF1R, HSP90AA1, MAPK8, PIK3CA, ERBB2, KRAS, EGFR, MTOR
hsa05205	Proteoglycans in cancer	7	2.59E-07	ERBB3, PIK3CA, ERBB4, ERBB2, KRAS, EGFR, MTOR
hsa04010	MAPK signaling pathway	7	2.50E-06	CSF1R, MAPK8, ERBB3, ERBB4, ERBB2, KRAS, EGFR
hsa05212	Pancreatic cancer	6	6.38E-08	MAPK8, PIK3CA, ERBB2, KRAS, EGFR, MTOR
hsa01521	EGFR tyrosine kinase inhibitor resistance	6	7.74E-08	ERBB3, PIK3CA, ERBB2, KRAS, EGFR, MTOR
hsa05215	Prostate cancer	6	2.16E-07	HSP90AA1, PIK3CA, ERBB2, KRAS, EGFR, MTOR
hsa01522	Endocrine resistance	6	2.27E-07	MAPK8, PIK3CA, ERBB2, KRAS, EGFR, MTOR
hsa05226	Gastric cancer	6	1.80E-06	ABCB1, PIK3CA, ERBB2, KRAS, EGFR, MTOR
hsa05207	Chemical carcinogenesis - receptor activation	6	1.06E-05	HSP90AA1, PIK3CA, KRAS, CYP3A4, EGFR, MTOR
hsa05230	Central carbon metabolism in cancer	5	2.97E-06	PIK3CA, ERBB2, KRAS, EGFR, MTOR
hsa05210	Colorectal cancer	5	6.72E-06	MAPK8, PIK3CA, KRAS, EGFR, MTOR
hsa05231	Choline metabolism in cancer	5	1.12E-05	MAPK8, PIK3CA, KRAS, EGFR, MTOR
hsa05224	Breast cancer	5	5.50E-05	PIK3CA, ERBB2, KRAS, EGFR, MTOR
hsa04014	Ras signaling pathway	5	3.46E-04	CSF1R, MAPK8, PIK3CA, KRAS, EGFR
hsa05213	Endometrial cancer	4	8.55E-05	PIK3CA, ERBB2, KRAS, EGFR
hsa05221	Acute myeloid leukemia	4	1.31E-04	CSF1R, PIK3CA, KRAS, MTOR
hsa05223	Non-small cell lung cancer	4	1.62E-04	PIK3CA, ERBB2, KRAS, EGFR

The four investigated drugs exhibited the following degree-based ranking (descending order): Lapatinib (degree = 22), Neratinib (Agha et al., 2025), Trastuzumab (Xue et al., 2022), and Tucatinib (Song et al., 2021) (Table 6). Similarly, the target genes were ranked as follows: PIK3CA, MAPK8, ERBB3, CSF1R, EGFR,HSP90AA1, ABCB1, MCL1, ERBB2, ERBB4, CYP3A4, KRAS, and MTOR. These results collectively indicate that while all four drugs modulate tumor progression through multiple targets, the specific target profiles and underlying mechanisms exhibit both shared and distinct characteristics.

**TABLE 6:** Drugs and targets ranked by degree of the Drug-Target-Pathway network.

Drugs/Targets	Degree
Lapatinib	22
Neratinib	17
Trastuzumab	15
Tucatinib	13
PIK3CA	10
MAPK8	10
ERBB3	8
CSF1R	8
EGFR	7
HSP90AA1	7
ABCB1	7
MCL1	6
ERBB2	6
ERBB4	6
CYP3A4	4
KRAS	4
MTOR	4

#### 3.2.4 Molecular docking

**FIGURE 4:**
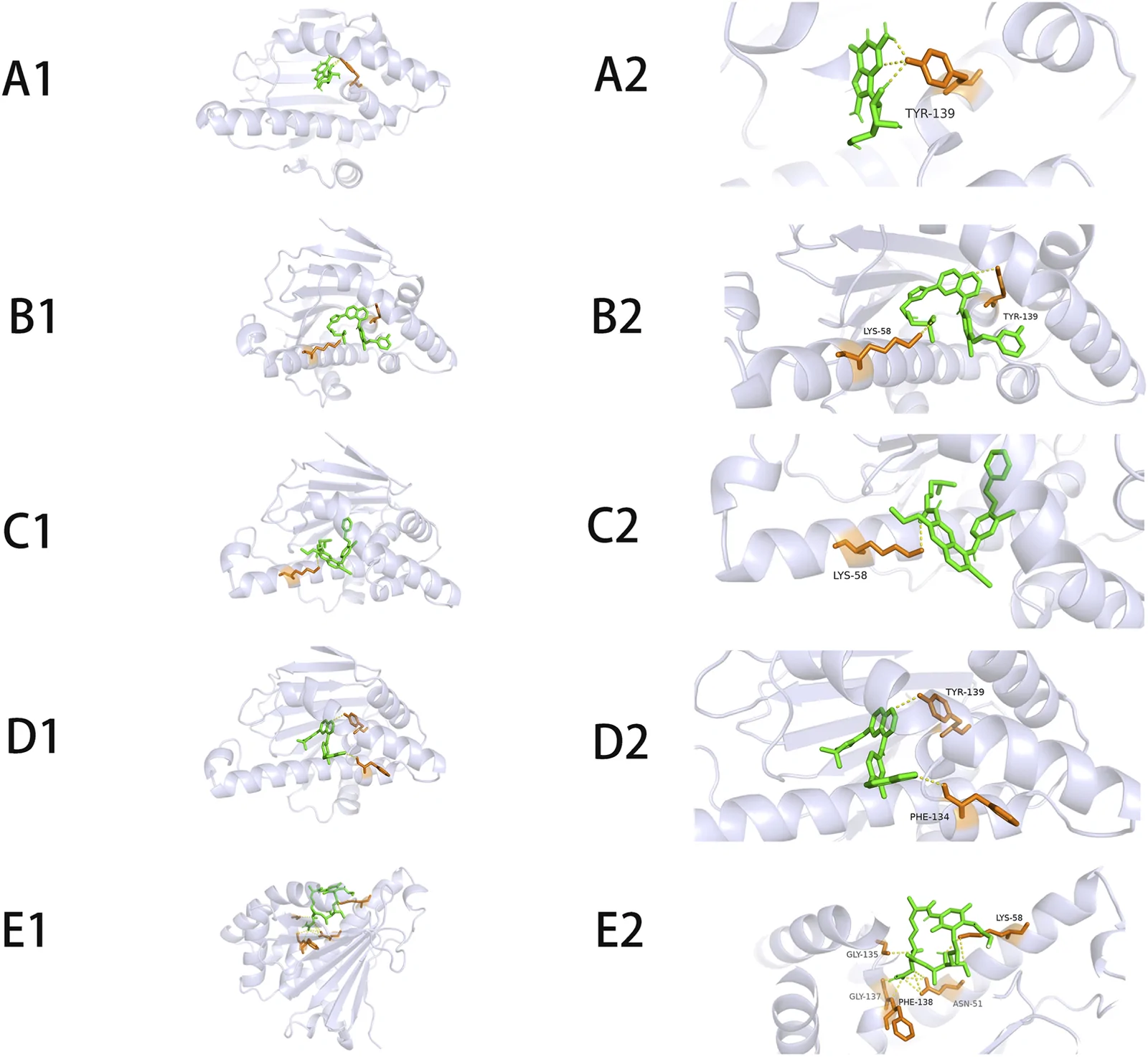
Molecular docking between HSP90AA1 and the drugs. (A1) The binding mode of HSP90AA1 and trastuzumab. (B1) The binding mode of HSP90AA1 and lapatinib. (C1) The binding mode of HSP90AA1 and neratinib. (D1) The binding mode of HSP90AA1 and tucatinib. (E1) The binding mode of HSP90AA1 and tanespimycin. (A2), (B2), (C2), (D2) and (E2) represents the enlarged picture of HSP90AA1 and drugs binding, in which the dotted line represents the hydrogen bond, the text part represents the residue name, and the lower right corner represents the atom binding diagram of molecule and protein.

To validate the findings from network pharmacology, molecular docking was employed to evaluate the binding affinities between the screened drugs and target proteins. Based on degree values derived from Cytoscape’s MCODE and CytoHubba algorithms, as well as the Drug-Target-Pathway network analysis, HSP90AA1 was identified as a key target gene. In addition to the four candidate drugs, we included Tanespimycin, a known HSP90AA1 inhibitor, for comparative analysis. As presented in Figure 4, molecular docking simulations revealed stable binding conformations between HSP90AA1 and all five investigated compounds. Our analysis demonstrated the formation of significant hydrogen bonds between HSP90AA1 and these drugs (Table 7), indicative of strong and stable interactions. Notably, Tucatinib exhibited the lowest binding energy (−9.6 kcal/mol), representing the most thermodynamically favorable interaction, followed by Lapatinib (−9.2 kcal/mol) and Neratinib (−7.8 kcal/mol). In contrast, Tanespimycin showed relatively higher binding energy (−6.9 kcal/mol). These results collectively confirm the spontaneous binding capability of HSP90AA1 with these therapeutic agents, with Tucatinib demonstrating particularly strong molecular interactions.

**TABLE 7:** Molecular docking analysis of HSP90AA1 and the drugs.

Core target	Ligand	Binding energy (kcal/mol)
HSP90AA1	Trastuzumab	−7.3
	Lapatinib	−9.2
	Neratinib	−7.8
	Tucatinib	−9.6
	Tanespimycin	−6.9

#### 3.2.5 Molecular dynamic simulation

##### 3.2.5.1 Root mean square deviation (RMSD) analysis

**FIGURE 5:**
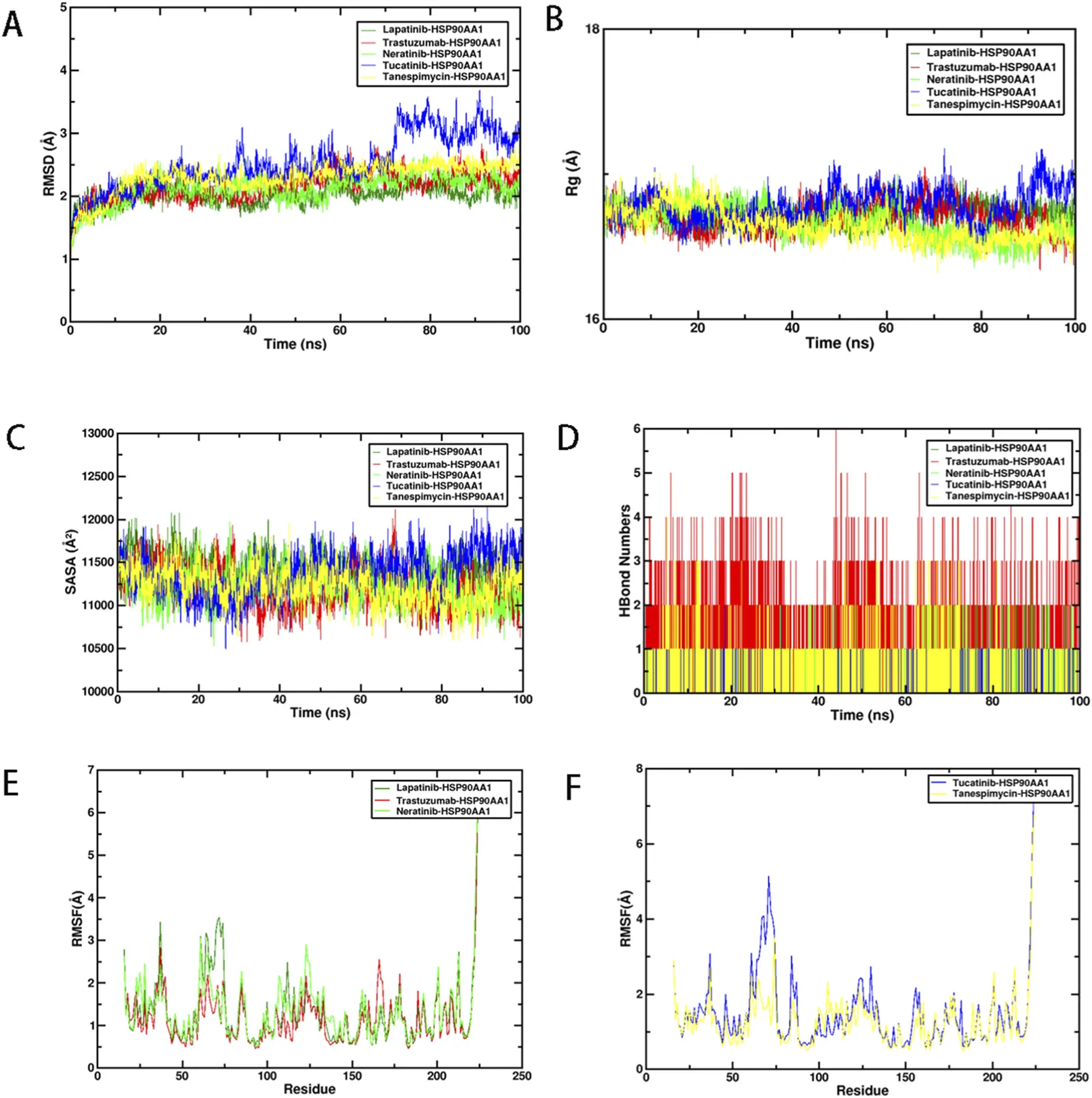
Molecular dynamics simulation trajectory plot of protein-ligand complexes during 100ns MD simulation. **(A)** The RMSD plot illustrated the dynamic of protein during a simulation period lasting 100 ns? **(B)** Radius of gyration value (Rg). **(C)** Solvent accessible surface area (SASA). **(D)** Hydrogen bonds. **(E)** RMSF visualisation of Trastuzumab-HSP90AA1 complex, Lapatinib-HSP90AA1 complex, and Neratinib-HSP90AA1 complex. **(F)** RMSF visualisation of Tucatinib-HSP90AA1 complex and Tanespimycin-HSP90AA1 complex.

In molecular dynamics simulations, the RMSD values, which quantify the deviation of atomic configurations from their initial states, serve as a robust metric for assessing the stability of the system. Four systems (Trastuzumab-HSP90AA1 complex, Lapatinib-HSP90AA1 complex, Neratinib-HSP90AA1 complex, and Tanespimycin-HSP90AA1 complex) reached equilibrium after 10 ns, with RMSD fluctuations stabilized at 2.4 Å, 2.2 Å, 2.2 Å and 2.4 Å, respectively (Figure 5A). The Tucatinib-HSP90AA1 complex exhibited noticeable conformational adaptations during the initial 0–70 ns of the trajectory, which is attributed to the complex induced-fit adjustments of the ligand within the binding pocket. Subsequently, the system stabilized and maintained a steady plateau at approximately 3.2 Å from 70 to 100 ns (Figure 5A). This profile indicates that despite a higher equilibrium baseline compared to the other more rigid complexes (2.2–2.4 Å), reflecting the structural flexibility inherent to tucatinib’s binding mode, the complex successfully achieved a reliable dynamic equilibrium without structural dismantling.

##### 3.2.5.2 Root mean square fluctuation (RMSF) analysis

RMSF analysis is a vital tool for assessing subtle variations within the protein chain during simulations, reflecting the fluctuations of amino acid residues. The average RMSF values were recorded as 1.3 Å, 1.2 Å, 1.3 Å, 1.5Å and 1.3 Å for the Trastuzumab-HSP90AA1 complex, Lapatinib-HSP90AA1 complex, Neratinib-HSP90AA1 complex, Tucatinib-HSP90AA1 complex and Tanespimycin-HSP90AA1 complex (Figure 5B). Based on these RMSF values, these complexes demonstrated the stable behavior. In RMSF graphs, Prominent peaks in the graphs indicate regions of the protein experiencing frequent and continuous fluctuations during the simulation. These residues are part of the protein active site and contribute to the dynamic binding interactions with proteins. Typically, terminal and loop sections exhibit greater variability than secondary structures like alpha-helices and beta-strands.

##### 3.2.5.3 Radius of gyration (Rg) analysis

Rg provides a measure of the overall compactness and size of the complex, offering insights into the conformational changes that occur upon complex formaton. The Rg from the simulation trajectories indicated that a larger Rg corresponds to a more mobile complex, while a smaller Rg reflects a more rigid structure. The Rg of these complexes equilibrated at 16.7 Å, 16.7 Å, 16.7 Å, 16.8 Å, and 16.6 Å, respectively, indicating that the complexes maintain compact overall structures (Figure 5C).

##### 3.2.5.4 Solvent accessible surface area analysis (SASA)

Solvent accessible surface area (SASA) assesses the area of a molecular surface that can be contacted by solvent. An increase in SASA values in the curve indicates more exposed hydrophobic residues, which may be related to protein flexibility or structural changes (Figure 5D).

##### 3.2.5.5 Hydrogen bond analysis

Protein-ligand complexes heavily depend on hydrogen bonding to maintain their secondary structure. The number of hydrogen bonds in the Lapatinib-HSP90AA1 and Tucatinib-HSP90AA1 mainly fluctuated between 0 and 1 (Figures 5E,F). The SASA values of the Trastuzumab-HSP90AA1 complex mainly fluctuated between 0 and 4 (Figure 5E), the Neratinib-HSP90AA1 complex between 0 and 2 (Figure 5E), and the Tanespimycin-HSP90AA1 complex between 0 and 3 (Figure 5F).

### 3.3 Comprehensive analysis of HSP90AA1 Expression and Clinical Significance in Breast Cancer

**FIGURE 6:**
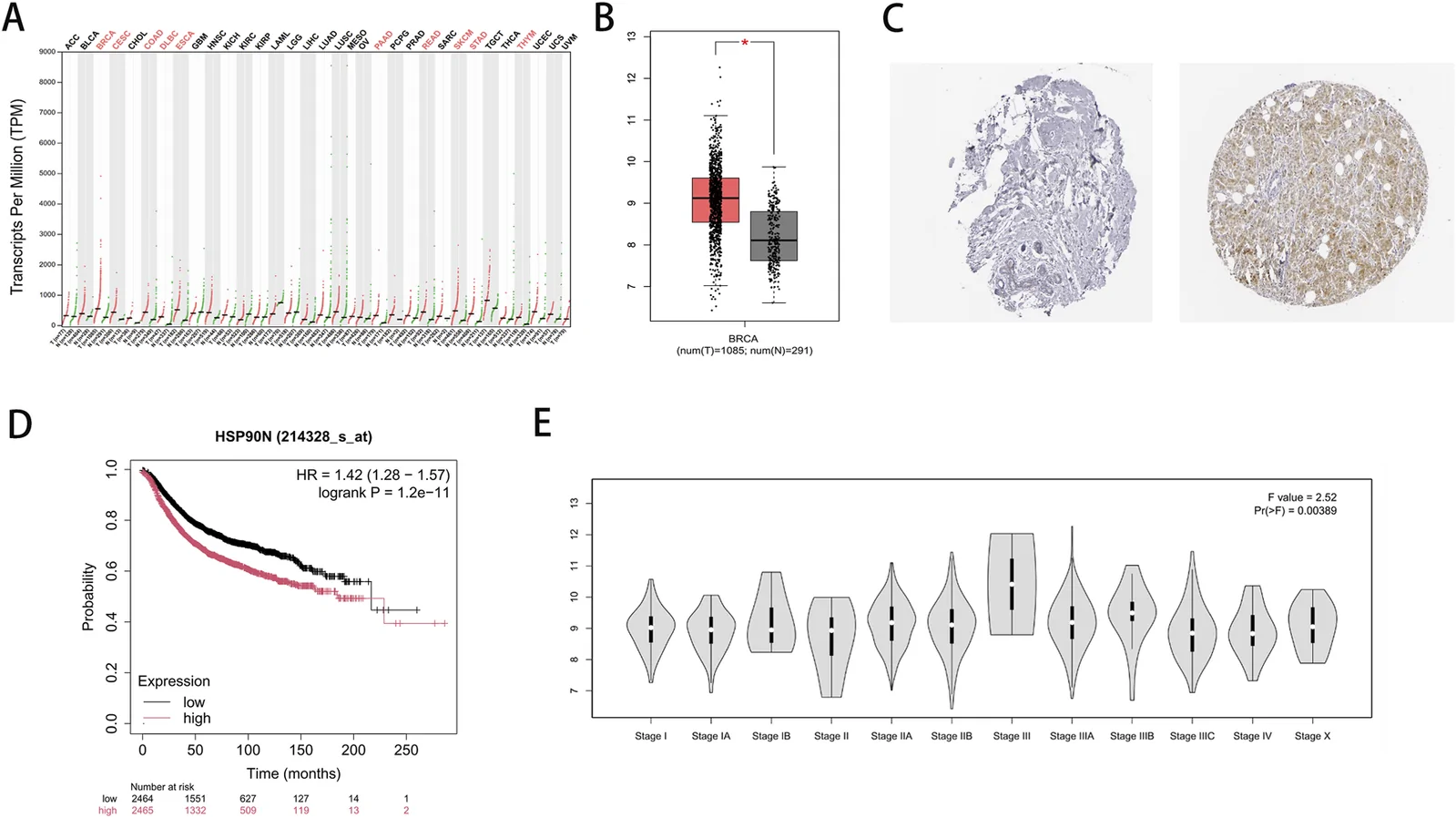
HSP90AA1 Expression and Clinical Significance in Breast Cancer. **(A)** HSP90AA1 expression between tumor and normal tissues in multiple cancer types based on the GEPIA database. **(B)** Evaluation of HSP90AA1 protein levels in breast tumors and normal tissues using the GEPIA database. **(C,D)** HIC results for HSP90AA1 in breast tumors and normal tissues were analyzed using the HPA database. **(E)** The impact of HSP90AA1 expression level on prognosis was analyzed based on the KM PLOTTER database. **(F)** The GEPIA database was utilized to assess the expression level of HSP90AA1 across different stages of breast cancer. All the data analysis. *p < 0.05.

The expression profiles of HSP90AA1 across diverse tissues were initially evaluated via the Gene Expression Profiling Interactive Analysis (GEPIA) database. As demonstrated in Figure 6A, HSP90AA1 exhibited significant differential expression between tumor and normal tissues in multiple cancer types, including breast cancer, cervical squamous cell carcinoma, colon adenocarcinoma, diffuse large B-cell lymphoma, esophageal cancer, pancreatic adenocarcinoma, rectal adenocarcinoma, skin cutaneous melanoma, stomach adenocarcinoma and thymic tumors.

Notably, HSP90AA1 expression was markedly upregulated in breast cancer compared to normal tissue (Figure 6B), a finding corroborated by immunohistochemical (IHC) data derived from the Human Protein Atlas (HPA) database (Figures 6C,D). Survival analysis utilizing the KM Plotter database indicated that elevated HSP90AA1 expression correlated with diminished overall survival in patients with breast cancer (Figure 6E). Furthermore, GEPIA2.0 analysis demonstrated a significant association between HSP90AA1 expression levels and advanced clinical stages of breast cancer (Figure 6F). These data indicate that aberrant HSP90AA1 overexpression in breast cancer tissues is significantly associated with an unfavorable clinical prognosis.

### 3.4 HSP90AA1 promotes cell proliferation, cell migration and invasion

**FIGURE 7:**
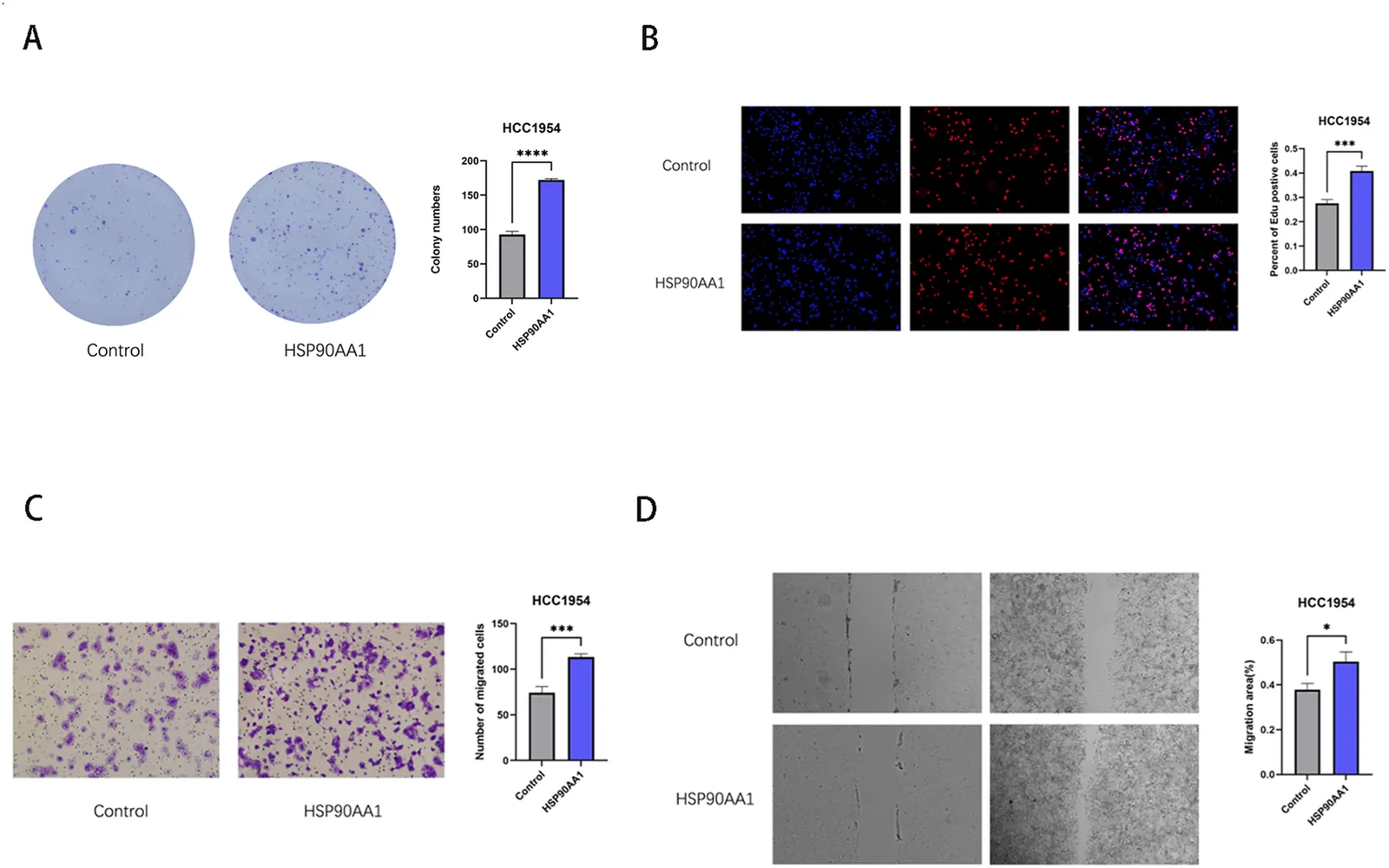
HSP90AA1 promotes cell proliferation and invasion. The cell proliferation assays and invasion assays were conducted, including, colony formation assay **(A)**, EdU assay **(B)**, transwell assay **(C)**, scratch assay **(D)**. All experiments were repeat three times (n = 3), compared variances using F test and analyzed using an unpaired t-test, error bars represent SD. *p < 0.05; ***p < 0.001; ****p < 0.0001.

To evaluate the functional role of HSP90AA1 in breast cancer progression, HCC1954 cells overexpressing HSP90AA1 were established. Compared to the control group, cells overexpressing HSP90AA1 exhibited significantly enhanced colony formation efficiency (Figure 7A), accelerated proliferation rates, and an increased percentage of EdU positive cells (Figure 7B), indicating that HSP90AA1 drives tumor cell growth. Furthermore, the overexpression of HSP90AA1 conferred a more aggressive phenotype in Transwell invasion (Figure 7C) and wound healing assays (Figure 7D). Collectively, these findings confirm that HSP90AA1 promotes the proliferation, migration, and invasion of breast cancer cells *in vitro*. Consistent with the HCC-1954 results, HSP90AA1 overexpression in BT474 cells significantly increased colony formation (Supplementary Figure S1A) and enhanced migratory capacity in Transwell assays (Supplementary Figure S1B). This cross-validation confirms that HSP90AA1 consistently drives an aggressive malignant phenotype across diverse HER2-positive backgrounds.

### 3.5 Targeting HSP90AA1 with tanespimycin in combination with trastuzumab and lapatinib significantly modulates the PI3K/Akt/mTOR signaling pathway and promotes apoptosis

**FIGURE 8:**
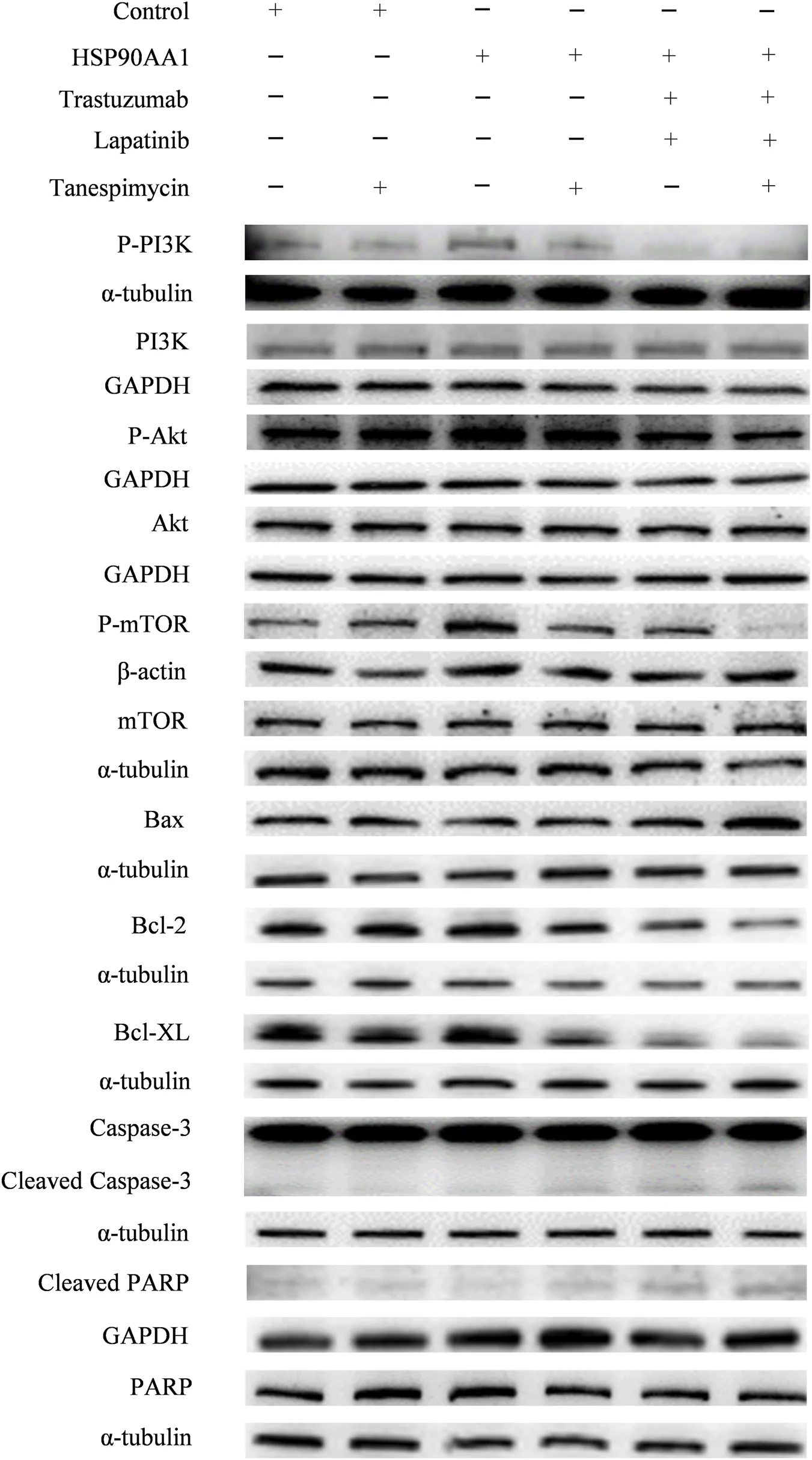
Western blot analysis assessing the expression and phosphorylation of PI3K, Akt, and mTOR, along with key Apoptotic markers in different treatment groups.

Western blot analysis was utilized to assess the regulatory impact of HSP90AA1 expression and targeted intervention on survival signaling and the apoptotic program in HCC1954 cells (Figure 8). The results indicated that monotherapy with tanespimycin yielded no significant alterations in the phosphorylation status of PI3K, Akt, or mTOR. Conversely, overexpression of HSP90AA1 markedly upregulated the phosphorylation levels of PI3K, Akt, and mTOR (p-PI3K, p-Akt, and p-mTOR) compared to the control group, suggesting that HSP90AA1 potentiates oncogenic signaling transduction. Furthermore, HSP90AA1 overexpression disrupted the apoptotic equilibrium, characterized by a reduction in the levels of the pro-apoptotic protein Bax and the execution markers Cleaved Caspase-3 and Cleaved PARP, concomitant with a significant elevation in the anti-apoptotic proteins Bcl-2 and Bcl-XL.

Notably, in cells overexpressing HSP90AA1, the triple combination therapy comprising tanespimycin, trastuzumab, and lapatinib effectively reversed these effects. The combination treatment not only significantly suppressed the phosphorylation of PI3K, Akt, and mTOR but also effectively restored apoptotic sensitivity. Specifically, the expression of Bax was significantly increased, whereas the levels of Bcl-2 and Bcl-XL were markedly attenuated in the combination group. Correspondingly, the levels of Cleaved Caspase-3 and Cleaved PARP, acting as the final executioners of the apoptotic cascade, were significantly elevated following combination therapy, although the total protein levels of Caspase-3 and PARP remained largely unchanged. Collectively, these data confirm that the combination therapy induces cell death in HER2-positive breast cancer cells by inhibiting the PI3K/Akt/mTOR signaling axis and reactivating the apoptotic machinery.

To provide robust, direct functional evidence of apoptosis corroborating our molecular marker profiling, we performed TUNEL fluorescence assays across all treatment groups. As depicted in Supplementary Figure S1C, while monotherapies induced limited apoptosis, the triple-combination regimen (Trastuzumab + Lapatinib + Tanespimycin) maximized the percentage of TUNEL-positive cells in HSP90AA1-overexpressing HCC1954 cells, confirming profound apoptotic execution.

Furthermore, to address the clinical context wherein anti-HER2 therapies are typically co-administered with chemotherapy, we evaluated our novel regimen against current standard clinical practices. We conducted a comparative TUNEL assay in wild-type HCC1954 cells among three distinct cohorts (Bray et al., 2024): Paclitaxel alone (Guo et al., 2021), the current first-line clinical standard (Paclitaxel + Trastuzumab + Pertuzumab), and (Yang et al., 2022) our proposed non-chemotherapy triple regimen (Trastuzumab + Lapatinib + Tanespimycin). Strikingly, the results (Supplementary Figure S1D) revealed that our targeted triple-combination achieved an apoptotic efficacy that is highly comparable to, and statistically robust against, the conventional dual-targeted chemotherapy regimen. This direct comparison unequivocally validates the potent cytotoxicity of the HSP90AA1-targeted combination, even in the absence of traditional cytotoxic chemotherapy agents.

## 4 Discussion

The treatment of HER2-positive breast cancer has advanced significantly with the introduction of targeted therapies; however, challenges such as drug resistance and adverse events remain substantial. This study systematically evaluated the pharmacovigilance profiles and underlying molecular mechanisms of trastuzumab monotherapy versus combinatorial regimens involving lapatinib, neratinib, and tucatinib by integrating real world data from the FDA Adverse Event Reporting System (FAERS) with advanced computational analyses. Compared to previous studies that mainly focused on trastuzumab monotherapy or single combinations, this study is the first to integrate FAERS-based safety analysis with network pharmacology, docking, molecular dynamics and *in vitro* validation, covering several trastuzumab-containing regimens. This multimodal design extends prior findings by linking regimen-specific AE spectra to shared molecular mechanisms centered on HSP90AA1 and PI3K/Akt/mTOR signaling.

Our retrospective pharmacovigilance analysis of the FAERS database revealed distinct and divergent adverse event (AE) profiles among the four treatment regimens. To our knowledge, this study represents the first systematic comparison within FAERS of trastuzumab monotherapy versus its combination with three TKIs, elucidating the real-world adverse event spectrum, time of onset, and Weibull risk models. Trastuzumab monotherapy was associated with a higher proportion of AEs related to cardiac dysfunction and tumor progression, such as decreased ejection fraction (Guglin et al., 2019) and breast cancer recurrence (Olson et al., 2013). Trastuzumab primarily targets the extracellular domain of HER2 (Cordero et al., 2022), although effective, resistance mechanisms often lead to disease progression (Lu et al., 2021). In contrast, combination therapies, particularly those involving oral tyrosine kinase inhibitors (TKIs) such as lapatinib, neratinib, and tucatinib, exhibited unique AE profiles. The high incidence of dermatologic and gastrointestinal disorders (Yu et al., 2022; Jacob et al., 2019), especially diarrhea, in regimens containing TKIs is well documented in clinical trials and reflects the off-target effects of pan- or selective HER family inhibition (Bielec et al., 2020). While AE profiles specific to the combination of tucatinib and trastuzumab remain less comprehensively reported, a multicenter, randomized, placebo controlled phase III trial (Murthy et al., 2020) evaluating tucatinib in combination with trastuzumab and capecitabine in patients with HER2-positive advanced breast cancer (including those with brain metastases) indicated that peripheral neuropathy was reported in approximately 11.6% of patients receiving the regimen containing tucatinib. Notably, although combination therapies carry a theoretical risk of increased toxicity, the observed incidence of AEs was substantially lower than that associated with monotherapy. This observation may stem from several factors, including the relatively smaller sample size of patients receiving combination therapy within the FAERS database. Alternatively, a more plausible mechanism may involve synergistic pharmacological interactions that modulate the overall safety profile, potentially by enabling reduced dosages or shorter treatment durations for individual agents. These findings warrant further validation in large-scale prospective studies.

Analyzing the temporal dynamics of adverse events via the Weibull distribution model offers critical insights for clinical management. The median time-to-onset (TTO) of adverse events was significantly prolonged for all combinatorial regimens compared to trastuzumab monotherapy. This observation holds substantial clinical implications, suggesting that monitoring strategies should be tailored to specific regimens. Furthermore, it underscores the necessity for intensified patient surveillance during the initial treatment phase to mitigate early onset AEs. However, variations in the β-values among the combination regimens also reveal subtle differences in their temporal risk profiles. The lapatinib combination regimen demonstrated the lowest β-value, indicating a more pronounced concentration of risk in the early phase, potentially attributable to rapid EGFR pathway inhibition by lapatinib leading to the swift onset of adverse events such as diarrhea and skin rash (Rasheed et al., 2007).

Compared to previous studies that focused solely on network pharmacology, this study innovatively integrates FAERS safety data with a multimodal framework to identify HSP90AA1 as a shared target, revealing for the first time the core role of HSP90AA1 in combination therapy. Despite the divergent adverse event profiles documented in the FAERS database, our systems pharmacology approach identified HSP90AA1 as a unifying molecular target. Toxicities specific to individual agents, such as the prominent signal of diarrhea in combination therapies identified in our FAERS analysis, are largely attributable to unique off-target profiles, exemplified by concurrent EGFR inhibition in the gastrointestinal tract by lapatinib and neratinib (Borges et al., 2018). Herein lies the critical conceptual nexus between our FAERS safety evaluation and the subsequent molecular investigation. While the clinical toxicity profiles inevitably diverge due to these off-target effects, we found that the conserved anti-tumor efficacy of these regimens converges substantially upon their shared modulation of the HSP90AA1 chaperone system and its downstream signaling networks. By identifying this shared molecular vulnerability, our study aims to answer the clinical dilemma posed by the FAERS data: how to maintain efficacy while reducing toxicity.

HSP90AA1, an inducible isoform of the heat shock protein 90 family (Kim et al., 2021), exhibits significant overexpression in the tumor microenvironment (Wang et al., 2019), where neoplastic cells experience perpetual proteotoxic stress induced by hypoxia, nutrient scarcity, acidosis, and genomic instability. Functioning as an ATP-dependent molecular chaperone, HSP90AA1 facilitates oncogenic transformation by stabilizing mutated and overexpressed client proteins (Klemke et al., 2021), with HER2 constituting a particularly dependency-prone client (Kang et al., 2022). Moreover, through its stabilization of pivotal signaling components in the PI3K/Akt, RAS/RAF/MEK/ERK, and other survival pathways (Yin et al., 2021), HSP90AA1 enables tumor cells to evade apoptosis and acquire resistance to conventional chemotherapeutics, radiation, and targeted agents. Our data further reveal that the antitumor mechanism of the triple combination therapy (incorporating the HSP90AA1 inhibitor tanespimycin) extends beyond simple signal blockade to the restoration of apoptotic competency. Aberrant activation of the PI3K/Akt/mTOR pathway typically facilitates evasion of cell death by phosphorylating and inactivating pro-apoptotic proteins such as Bax (Zhang et al., 2021). In our model, the triple combination not only abrogated the upstream PI3K/Akt/mTOR phosphorylation cascade but, critically, shifted the rheostat of Bcl-2 family proteins. The downregulation of Bcl-2 and Bcl-XL relieves their inhibition of Bax, leading to mitochondrial outer membrane permeabilization, which subsequently triggers the proteolytic activation of Caspase-3 and the cleavage of PARP (Ranjan et al., 2020). The significant elevation of cleaved PARP, a hallmark of compromised DNA repair and irreversible apoptosis, confirms that the combination regimen successfully converts cytostatic effects into overt cytotoxicity. These findings provide a solid molecular basis for overcoming the apoptotic evasion mechanisms common in HER2-positive breast cancer. Our comparative TUNEL assays indicate that this targeted triple-blockade induces an apoptotic rate comparable to that of standard paclitaxel-based dual-targeted therapy *in vitro*. This observation highlights a promising translational potential: exerting robust anti-tumor effects through a purely targeted mechanism, which may eventually offer a viable alternative for patients who are unsuitable candidates for conventional cytotoxic chemotherapy.

Mutations in the *PIK3CA* gene and loss or inactivation of PTEN are the primary causes of aberrant PI3K/Akt signaling pathway activation (Chen et al., 2017). Aberrant activation of the PI3K/Akt signaling pathway is a key mechanism of resistance in HER2-positive malignancies (Bao et al., 2020). When the PI3K/Akt pathway is aberrantly activated, tumor cells can bypass HER2 inhibition and maintain proliferation and survival through sustained signaling. Subsequently, activated Akt phosphorylates downstream substrates, including the mammalian target of rapamycin (mTOR), to drive protein synthesis, cell growth, and proliferation (Alsolmei et al., 2019). Concurrently, Akt suppresses apoptosis by phosphorylating and inactivating pro-apoptotic proteins, enabling tumor cells to evade cell death and thereby conferring resistance to HER2-targeted therapies (Zhao Z. et al., 2021).

Clinical studies have revealed that a subset of patients with HER2-positive breast cancer exhibits primary resistance to trastuzumab, while a substantial proportion acquires secondary resistance during treatment (Zazo et al., 2016). Lapatinib, a dual tyrosine kinase inhibitor targeting both HER1 and HER2 (Gibson et al., 2015), is utilized for patients with HER2-positive breast cancer who have developed resistance to trastuzumab (Chumsri et al., 2022). However, with prolonged administration, the emergence of resistance to lapatinib itself poses a significant clinical challenge (McDermott et al., 2019). A study investigating lapatinib plus capecitabine in patients with HER2-positive advanced breast cancer who had experienced failure of prior trastuzumab-based therapy demonstrated that the wild-type PIK3CA cohort had a significantly greater progression-free survival (PFS) benefit compared to patients with PIK3CA mutations (Takano et al., 2018). These findings underscore that PIK3CA status, a critical biomarker of the PI3K/Akt pathway, is intrinsically linked to treatment response and resistance development in HER2-positive breast cancer. Analysis of clinical studies in patients with HER2-positive gastric cancer revealed a significant enrichment of PIK3CA mutations upon disease progression in those who developed resistance to trastuzumab (Zhang et al., 2023). In summary, the PI3K/Akt signaling pathway plays a critical role in mediating resistance to both chemotherapy and HER2-targeted agents in HER2-positive tumors.

Tanespimycin (17-Allylamino-17-demethoxygeldanamycin, 17-AAG) is an inhibitor of heat shock protein 90 (HSP90). Mechanistically, it binds specifically to HSP90, thereby disrupting the intrinsic chaperone function of the protein (Li et al., 2020). This inhibition subsequently leads to the ubiquitin-mediated degradation and functional inactivation of a suite of client proteins that rely on HSP90 for their stability and conformational maturation. Multiple clinical trials investigating tanespimycin have been conducted specifically for HER2-positive tumors. In the field of breast cancer, several studies explored combinations of tanespimycin with other therapeutic agents. A phase II study evaluating tanespimycin combined with trastuzumab in patients with advanced HER2 positive breast cancer refractory to trastuzumab demonstrated significant antitumor activity for this dual regimen (Modi et al., 2011). Another study demonstrated that conjugating the trastuzumab antibody to the surface of micelles co-loaded with tanespimycin and paclitaxel, thereby integrating active targeting mechanisms, resulted in significantly enhanced anti-tumor efficacy against HER2-positive breast cancer, with no signs of acute toxicity and significantly improved treatment tolerability (Soni et al., 2017). In other oncology fields: A phase 2 clinical trial (NCT00779428) evaluated the efficacy and safety of tanespimycin in combination with bortezomib for the treatment of relapsed or refractory multiple myeloma. In terms of efficacy, the combination therapy may achieve tumor remission in some relapsed/refractory patients and showed good tolerability (Richardson et al., 2011). A clinical trial of docetaxel and tanespimycin for the treatment of adult solid tumors also achieved tumor remission or stabilization and good tolerability (Iyer et al., 2012). However, due to mergers and acquisitions and strategic pipeline adjustments in the pharmaceutical industry, the further development of this drug has been put on hold, so tanespimycin has not yet been used in routine clinical practice. Although large-scale Phase III clinical trials directly evaluating tanespimycin as monotherapy or in combination with standard targeted therapies in HER2-positive tumors remain limited, this study aims to highlight the exploratory value of triple therapy (trastuzumab + lapatinib + tanespimycin), targeting HSP90AA1 to regulate HER2-positive malignancies through the PI3K/Akt pathway. This approach aims to achieve synergistic efficacy while reducing toxicity, highlighting a promising therapeutic strategy with the potential to overcome resistance to HER2-directed treatments. Further Phase III trials are needed to verify tolerability before it can be used in clinical practice.

Furthermore, a critical challenge in translating our findings to clinical practice lies in the non-invasive monitoring of HSP90AA1 status in patients. Traditional tissue biopsies are invasive and often fail to capture the spatiotemporal heterogeneity of metastatic lesions. In contrast, liquid biopsy offers a promising solution to this dilemma. Sui et al. (2025) recently provided a comprehensive review of *in vivo* and *in vitro* circulating tumor cell (CTC) detection strategies (Sui and Zhang, 2025). They highlighted that emerging technologies, such as *in vivo* flow cytometry (IVFC) and functionalized intravenous catheter capture devices, can overcome the blood volume limitations of traditional *in vitro* assays, enabling high-sensitivity enrichment and real-time dynamic monitoring of rare CTCs. Drawing upon these insights, future clinical strategies could employ these advanced liquid biopsy techniques to isolate CTCs and quantify their HSP90AA1 expression levels. Just as PD-L1 expression on CTCs serves as a predictor for immunotherapy efficacy, HSP90AA1 levels on CTCs could serve as a non-invasive companion diagnostic biomarker. This strategy would facilitate the precise identification of HER2-positive patients with high HSP90AA1 abundance who are most likely to benefit from our proposed triple-combination regimen (Trastuzumab + Lapatinib + Tanespimycin), thereby overcoming resistance while optimizing clinical decision-making.

Despite the robust findings of this study, several limitations should be acknowledged. First, although the FDA Adverse Event Reporting System (FAERS) database serves as a valuable source of real-world evidence, it is still subject to reporting bias. As a passive monitoring system reliant on voluntary submissions, the database is prone to underreporting, with estimates suggesting that only 1%–10% of severe adverse events are captured (Goto et al., 2025), which may lead to an underestimation of risks and impact the generalizability of findings, particularly for milder or less recognized events. Furthermore, the lack of a denominator hinders accurate calculation of event rates, limiting the analysis to signal detection rather than absolute risk assessment. This may amplify signals for drugs with higher market penetration, leading to potential overinterpretation (Hoffman et al., 2014a). As a spontaneous reporting system, FAERS structurally lacks vital longitudinal parameters, including the precise distribution of medication duration, discontinuation rates, and explicit reasons for data censoring. Furthermore, incomplete documentation of comprehensive baseline clinical markers (such as baseline liver and kidney function) makes it inherently impossible to systematically control for these crucial confounding variables. Regarding Time-to-Onset (TTO) data, while adjusting for covariates like age, gender, and reporting year via Cox proportional hazards regression is standard practice in clinical cohorts, this approach requires a defined population with strict follow-up times for both events and non-events (censored data). Because FAERS exclusively captures patients who experienced an adverse event (numerator data) without tracking the unexposed or event-free population (denominator data), executing true survival analyses like Cox regression is mathematically unfeasible. Concurrent treatments introduce confounding factors, obscuring the causal relationship between specific drugs and adverse events, especially in polytherapy groups with smaller sample sizes, potentially exacerbating bias (Onda et al., 2023). Additionally, disproportionate analysis identifies statistical associations rather than establishing causality, and incomplete documentation of patient demographics, comorbidities, and concomitant medications further complicates these issues (Hoffman et al., 2014b). While these limitations may result in an underestimation or confounding of signal strength, they do not undermine the contribution of this study in identifying potential drug safety signals, as FAERS offers real-world insights that randomized controlled trials cannot capture. To mitigate these issues, we employed stratified analysis, exclusion criteria, and cross-referencing of other data sources to enhance the reliability of our results. Future research should validate these FAERS-derived signals through prospective observational registries or well-designed cohort studies and integrate multi-source data (e.g., electronic health records or clinical trial databases) to improve accuracy and causal inference, thereby strengthening pharmacovigilance practices. This will help overcome current limitations and drive more comprehensive drug safety evaluations (Hoffman et al., 2014a).

Secondly, while our computational tandem (molecular docking and 100-ns molecular dynamics simulations) successfully cross-verified the qualitative structural pairing between the selected drugs and HSP90AA1, certain computing limitations remain. Due to the macroscopic multi-platform scope of this study, our MD simulations focused primarily on evaluating structural integrity rather than exhaustive thermodynamic quantification. Consequently, multi-replicate trajectories and advanced binding free energy calculations (e.g., MM-PBSA), which are invaluable for precise thermodynamic profiling in dedicated computational biology investigations, were not performed. Future independent biophysical studies focusing strictly on the sub-atomic structural kinetics of these complexes are warranted to fully map their quantitative thermodynamic landscapes. Although our *in vitro* and *in silico* studies provide compelling evidence for the pivotal role of HSP90AA1, the absence of *in vivo* validation and direct clinical cohort validation remains a notable limitation of the current research. In real-world clinical practice, the extended treatment cycles for HER2-positive tumors and the invasive nature of obtaining serial matched tissue biopsies (pre- and post-treatment) pose significant logistical and ethical barriers to rapid patient cohort validation. Consequently, while our expression and survival analyses rely on large-scale public databases (e.g., GEPIA, KM Plotter, HPA) that offer robust statistical power, they cannot fully replace targeted, prospective clinical validation. We acknowledge that conventional cell culture models cannot fully recapitulate the complexities of the systemic tumor microenvironment, *in vivo* pharmacokinetics, and inter-organ crosstalk. Consequently, the direct clinical translation of our findings is inevitably constrained. Based on this, our conclusions should be strictly interpreted as an early proof-of-concept, rather than immediately actionable, practice-changing evidence. Future studies will focus on evaluating the efficacy and safety of the triple-combination regimen in patient-derived xenograft (PDX) or transgenic animal models to facilitate its translation into clinical practice.

Ultimately, while clinical trials remain essential for rigorously evaluating the efficacy and safety of such combination therapies, the primary significance of our current findings lies in their mechanistic and pharmacological implications. Although the proposed triple therapy demonstrates substantial mechanistic advantages in targeting the HSP90AA1-PI3K-Akt-mTOR axis, its pharmacological profile necessitates a careful evaluation of tolerability. Given that both lapatinib and tanespimycin are associated with gastrointestinal disturbances, hepatotoxicity, and fatigue, the concurrent administration of these agents at full overlapping doses poses a risk of cumulative toxicity (Roos et al., 2020; Shin et al., 2020). From a pharmacological perspective, however, the pronounced synergy observed in our study suggests a potential “dose-sparing” effect. This implies that effective inhibition of the PI3K-Akt-mTOR axis could be achieved at lower individual drug doses, thereby minimizing off-target toxicities (Janku et al., 2018).

## 5 Conclusion

This study establishes the first comprehensive safety profile of trastuzumab combined with TKIs derived from the FAERS database, elucidating toxicity patterns specific to each regimen and temporal risk characteristics to guide clinical monitoring. Mechanistically, integrated molecular docking and molecular dynamics simulations confirmed HSP90AA1 as a pivotal shared target mediating therapeutic efficacy. The triple regimen (comprising trastuzumab, lapatinib, and tanespimycin) effectively inhibits the PI3K/Akt/mTOR signaling axis, demonstrating the pharmacological and mechanistic validity of the HSP90AA1-targeted strategy in overcoming drug resistance in HER2-positive tumors. In conclusion, the multidimensional framework employed in this study deepens our mechanistic and pharmacological understanding of treatment strategies for HER2-positive breast cancer, laying a foundation for personalized risk stratification and the rational design of future combination therapies.

## Data Availability

The original contributions presented in the study are included in the article/Supplementary Material. The publicly available datasets analyzed in this study can be found in the FDA Adverse Event Reporting System (FAERS) database (https://www.fda.gov/drugs/drug-approvals-and-databases/fda-adverse-event-reporting-system-faers-database).
