# Supplementary material for: Trastuzumab-TKI combination in HER2-Positive tumors: a FAERS-Based safety profile and multimodal analysis of Tanespimycin’s role in enhancing targeting of the HSP90AA1-PI3K-Akt-mTOR axis
Source: Front Pharmacol. 2026 Aug 26;17:1844013. doi: 10.3389/fphar.2026.1844013 (PMC13555607; doi:10.3389/fphar.2026.1844013)
Supplement: Supplementary file 1 [file Supplementaryfile1.docx]

**Fig.S1** Validation of HSP90AA1-driven phenotypes in BT474 cells and TUNEL apoptosis assays. (A) Colony formation assay and (B) transwell invasion assay in BT474 cells, confirming that HSP90AA1 promotes cell proliferation and invasion across heterogeneous HER2+ cell lines. (C) Apoptosis was detected by TUNEL staining in HSP90AA1-overexpressing HCC1954 cells across six different treatment groups. The triple-combination therapy demonstrated the highest apoptotic execution. (D) Comparative TUNEL assay in wild-type HCC1954 cells evaluating Paclitaxel alone, standard clinical targeted chemotherapy (Paclitaxel + Trastuzumab + Pertuzumab), and the proposed triple regimen (Trastuzumab + Lapatinib + Tanespimycin). All experiments were independently repeated three times (n = 3). Statistical significance was determined using an unpaired t-test or one-way ANOVA as appropriate. Error bars represent SD. ns, not significant; *p < 0.05; **p < 0.01; ***p < 0.001; ****p < 0.0001.
